# Identification of valid reference genes for the normalization of RT qPCR gene expression data in human brain tissue

**DOI:** 10.1186/1471-2199-9-46

**Published:** 2008-05-06

**Authors:** David TR Coulson, Simon Brockbank, Joseph G Quinn, Suzanne Murphy, Rivka Ravid, G Brent Irvine, Janet A Johnston

**Affiliations:** 1 Division of Psychiatry and Neuroscience, School of Medicine and Dentistry, Queen's University Belfast, Whitla Medical Building, Belfast, BT9 7BL, Northern Ireland, UK; 2 Netherlands Brain Bank, Amsterdam, The Netherlands

## Abstract

**Background:**

Studies of gene expression in post mortem human brain can contribute to understanding of the pathophysiology of neurodegenerative diseases, including Alzheimer's disease (AD), Parkinson's disease (PD) and dementia with Lewy bodies (DLB). Quantitative real-time PCR (RT qPCR) is often used to analyse gene expression. The validity of results obtained using RT qPCR is reliant on accurate data normalization. Reference genes are generally used to normalize RT qPCR data. Given that expression of some commonly used reference genes is altered in certain conditions, this study aimed to establish which reference genes were stably expressed in post mortem brain tissue from individuals with AD, PD or DLB.

**Results:**

The present study investigated the expression stability of 8 candidate reference genes, (ubiquitin C [UBC], tyrosine-3-monooxygenase [YWHAZ], RNA polymerase II polypeptide [RP II], hydroxymethylbilane synthase [HMBS], TATA box binding protein [TBP], β-2-microglobulin [B2M], glyceraldehyde-3-phosphate dehydrogenase [GAPDH], and succinate dehydrogenase complex-subunit A, [SDHA]) in cerebellum and medial temporal gyrus of 6 AD, 6 PD, 6 DLB subjects, along with 5 matched controls using RT qPCR (TaqMan^® ^Gene Expression Assays). Gene expression stability was analysed using geNorm to rank the candidate genes in order of decreasing stability in each disease group. The optimal number of genes recommended for accurate data normalization in each disease state was determined by pairwise variation analysis.

**Conclusion:**

This study identified validated sets of mRNAs which would be appropriate for the normalization of RT qPCR data when studying gene expression in brain tissue of AD, PD, DLB and control subjects.

## Background

The mechanisms underlying certain neurodegenerative diseases such as Alzheimer's disease (AD), Parkinson's disease (PD), and dementia with Lewy bodies (DLB) remain poorly understood. One approach to further the understanding of such diseases is to study expression patterns of key genes in the affected tissue, human post-mortem brain. Quantitative real-time PCR (RT qPCR) is a fast, straightforward and reproducible technique which negates the need for post-PCR product handling and is increasingly becoming the method of choice for the accurate profiling of mRNA levels (gene expression) due to its accuracy, wide dynamic range and sensitivity [1-3]. RT qPCR enables the investigator to determine the expression levels of a given set of genes in a range of samples and is particularly useful when the sample quantity is limited [4-6].

Despite the many merits of RT qPCR, there are a number of inherent issues associated with its use, of which identification of a valid reference for data normalisation remains the most problematic [1,7]. At present, the most common method for such normalisation is the use of a single internal control reference gene – often referred to as a 'housekeeping gene'. The choice of genes regularly employed in RT qPCR for this purpose, such as β-actin, glyceraldehyde-3-phosphate dehydrogenase (GAPDH) and 18S rRNA stems from their use in traditional non- or semi-quantitative methods such as northern blotting. However, there is strong evidence in the literature to suggest that whilst the expression of some such reference genes may be constant under certain conditions, in other conditions they may fluctuate significantly [8-11]. There have been cases in which commonly accepted reference genes, such as GAPDH and β-actin, have been shown to be affected by *in vitro *experimental conditions [12], as well as clinical conditions such as asthma, and therefore may not always be suitable for normalisation [13]. Normalization of data using a non-validated reference gene may lead to inaccurate results and erroneous conclusions, and previous studies have reinforced the need to validate housekeeping genes prior to their use in a study [7,14].

The 'ideal' reference gene for RT qPCR would be one whose mRNA is consistently expressed at the same level in all samples under investigation, regardless of tissue type, disease state, medication or experimental conditions and would have expression levels comparable to that of the target [10]. However, the 'ideal' reference gene has yet to be discovered, and more than likely does not exist.

In addition to geNorm, several other Microsoft Excel based applications, such as NormFinder [15] and BestKeeper [16], are now available to asses the degree of variation in candidate reference genes. In geNorm, rather than using a single reference gene, Vandesompele *et al*. [17] proposed the use of more than one validated reference gene for data normalization. Use of two or more properly validated reference genes can provide improved resolution [18], and geNorm takes into account any differences in PCR reaction efficiencies, unlike earlier studies using the 2^-ΔΔCt ^method [19], which assumes all efficiencies to be at, or close to, 100%. To identify appropriate reference genes for a particular tissue and disease state, it is necessary to examine expression profiles of candidate genes to identify the most stable. Other studies have investigated this in post mortem brain tissue samples from individuals with other causes of death [20-22], and there is at least one study of reference gene expression in post mortem brain tissue from individuals with neurodegenerative conditions [23], but no previous investigations of post mortem brain tissue from individuals with AD, PD or DLB have used geNorm to identify the most stable reference genes, to our knowledge.

The issue of RNA quality is often raised in the context of gene expression analysis in human post mortem brain, as RNA integrity can be affected by pre- and post mortem factors [24]. An objective RNA integrity number (RIN, where 1 is poor, and 10 best) can now be determined using an Agilent Bioanalyser, replacing previous gel-based methods, and providing a better predictor of RNA quality [25]. The degree of RNA integrity required varies depending on the downstream RNA analysis method, and needs to be determined empirically for each application. This is an active area of research, and a recent study [26] analysed the influence of RNA sample RIN (1–10) on apparent gene expression levels and PCR efficiency, using RT qPCR. The study demonstrated that RT qPCR assay data based on short amplicons (70–250 bp) were independent of RIN. Longer amplicons (> 400 bp) were affected by RIN, and for these, it was recommended that samples with a RIN value of 5 or above were preferable [26]. The authors demonstrated that the impact of RNA integrity on relative quantification of gene expression could be minimized by normalization using validated internal reference genes, and by correcting for PCR efficiency [26].

In the present study, we examined the expression of candidate reference genes (Table 1) in the cerebellum and medial temporal gyrus from individuals with AD, PD, DLB, and controls, in order to provide a set of validated reference genes for use in the study of gene expression patterns in these neurodegenerative diseases.

**Table 1:** Candidate reference genes

**Gene Symbol**	**Gene Name**	**Cellular Function**	TaqMan Assay Number*	**Context Sequence**
**SDHA**	Succinate dehydrogenase complex, subunit A	dehydrogenase	Hs00417200_m1	CGCCGCCGTGGTCGAGCTAGAAAAT
**UBC**	Ubiquitin C	ubiquitination	Hs00824723_m1	CTGTGATCGTCACTTGACAATGCAG
**YWHAZ**	Tyrosine-3-monooxygenase	signal transduction	Hs00237047_m1	GGAGATAAAAAGAACATCCAGTCAT
**RP II**	RNA polymerase II polypeptide J	DNA-directed RNA polymerase	Hs00196523_m1	AACATCATTAAATCACAACTCCTAA
**HMBS**	Hydroxymethylbilane synthase	deaminase	Hs00609297_m1	GCGGCTGCAACGGCGGAAGAAAACA
**TBP**	TATA box binding protein	transcription factor	Hs99999910_m1	TGGGTTTTCCAGCTAAGTTCTTGGA
**B2M**	β-2-microglobulin	histocompatibility complex antigen	Hs99999907_m1	AAGTGGGATCGAGACATGTAAGCAG
**GAPDH**	Glyceraldehyde-3-phosphate dehydrogenase	dehydrogenase	Hs99999905_m1	GGCGCCTGGTCACCAGGGCTGCTTT

* 'm1' suffix denotes cDNA specific assays.

The 'context sequence' is the nucleotide sequence surrounding the region to which the probe binds. The primer and probe sequences for the TaqMan assays are not available. Detailed information for each TaqMan assay is freely available on

## Results

### RNA Integrity Analysis

Total RNA preparations were analysed using an Agilent 2100 Bioanalyzer (Agilent Technologies). Mean RIN values (± S.D.) were 3.2 ± 1.5 (range from 2.0 to 6.9), indicating that RNA degradation had occurred in some of these post mortem brain tissue samples, despite the efforts taken to minimise this (see Methods). RINs of repeat preparations of RNA from the same tissue block showed a high degree of correlation with previous RNA samples (linear regression analysis, r^2 ^= 0.66, p = 0.001) indicating that this was a feature of the tissue, rather than the RNA preparation method. We carried out some further analysis of this issue. Firstly, we found no correlation between RNA integrity (RIN) and disease progression, as indicated by sample Braak score (Spearman rank correlation). In addition, we found no significant correlations between RIN, and PMD or pH (Spearman rank correlation). We investigated whether any relationships existed between RIN, and gene expression data, expressed as the normalised relative quantity (Q) of each gene. There were no significant correlations between sample RIN and Q for any of the genes studied, in either cerebellum or medial temporal gyrus (Spearman rank correlation, p > 0.05 for all correlations). This indicated that the measures taken to minimise the impact of partial RNA degradation on the RT-qPCR assays (see below, in brief: inclusion of random nonomer primers in RT reactions in addition to oligo dT, and use of RT qPCR primer pairs designed to produce amplicons of 127 bp or less) had been successful. This is in agreement with the Fleige&Pfaffl study, where RT qPCR assays based on amplicons of 250 bp and less were independent of RNA quality [26].

### PCR Efficiency

cDNA was prepared from cerebellum and medial temporal gyrus of AD, PD, DLB, and matched control subjects. Using 10-fold serial dilutions of pooled cDNA, the PCR reaction efficiency of each gene assay, as listed in Table 1, was determined from the respective cDNA v C_t _efficiency plots (see Additional File 1). The efficiency plots for each of the candidate genes were found to have r^2 ^≥ 0.997. All gene assays were found to have efficiencies ≥ 92%, with several being ≥ 99% (Table 2).

**Table 2:** Efficiency data for candidate reference genes

**Gene Symbo**l	**Slope**	**R**^2^	**Efficiency**
**UBC**	-3.332	0.996	99%
**YWHAZ**	-3.345	0.998	99%
**RP II**	-3.412	0.9995	96%
**HMBS**	-3.294	0.997	100%
**TBP**	-3.317	0.9944	100%
**B2M**	-3.452	0.9992	95%
**GAPDH**	-3.519	0.999	92%
**SDHA**	-3.456	0.9985	95%

C_t _values obtained from 10-fold serial dilutions of pooled cDNA (from ×10 dilution to ×100000 dilution) were plotted against dilution factors. The reaction efficiency was calculated using the equation E = 10^(-1/slope) ^where 'E' is the efficiency and 'slope' is the gradient of the best fit line.

### Expression data analysis

**Figure 1:**
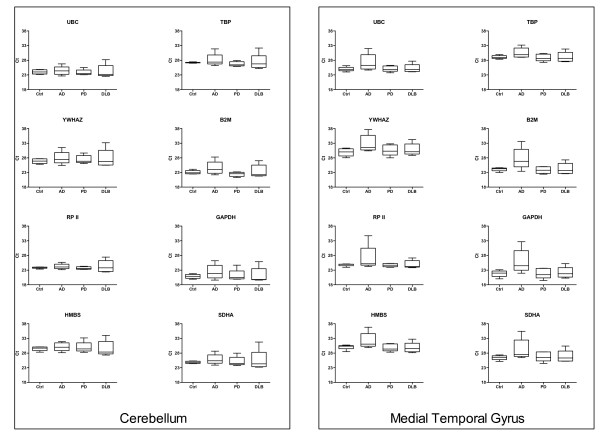
**RT qPCR cycle threshold value ranges for candidate genes**. Expression data displayed as cycle threshold (C_t_) values for each candidate reference gene in both cerebellum and medial temporal gyrus in controls, AD, PD and DLB subjects. The line in each box indicates the median. The box represents the 25^th ^and 75^th ^percentiles. Whiskers represent the maximum and minimum values.

mRNA levels of UBC, YWHAZ, RP II, HMBS, TBP, B2M, GAPDH and SDHA were determined in cerebellum and medial temporal gyrus from individuals with AD, PD, DLB and matched controls. The median, 25^th ^and 75^th ^percentiles, and range of C_t _values for each gene in each brain region across differing disease states are presented in Figure 1, illustrating the variation in expression levels between the different subject groups and brain regions. The standard deviation of gene-specific replicate samples was used to calculate the mean intra-run variation. The intra-run variation (CV) for each candidate reference gene ranged from 0.22% for UBC to 0.49% for GAPDH with a mean CV for all genes of 0.4%.

In order to quantify the degree of variation in candidate reference gene, and hence obtain a measure of the most stably expressed genes, the geNorm algorithm [17] was employed to analyse the raw expression data for our candidate genes. Using geNorm, it was possible to carry out sequential elimination of the least stable gene in any given experimental group, thus resulting in the exclusion of all but the two most stable genes in each case. In future gene expression studies we intend to measure levels of target genes in differing neurodegenerative disease subjects compared to control subjects; therefore it was necessary to ascertain the stability of the candidate genes across panels of grouped disease and control subjects, rather than in disease-specific subjects alone. geNorm analysis was performed on the gene expression data from the cerebellum and medial temporal gyrus of the following three groupings: controls and AD subjects, controls and PD subjects, and controls and DLB subjects. geNorm analysis of the candidate genes in each of the above groupings, in each brain region; identified sets of genes appropriate for normalization of data in each group.

To determine the optimal number of genes required for geometric mean normalization, geNorm calculates the pairwise variation (V_n_/V_n+1_) between sequential normalization factors (NF) (NF_n _and NF_n+1_). A large variation indicates that the gene included at that stage has a significant effect and should therefore be included for normalization. In the original publication describing geNorm [17] a threshold of 0.15 for the pairwise variation was established, below which the authors believe the inclusion of additional reference genes is not necessary; therefore we also adopted the same threshold as a cut-off for the inclusion of our references genes.

**Figure 2:**
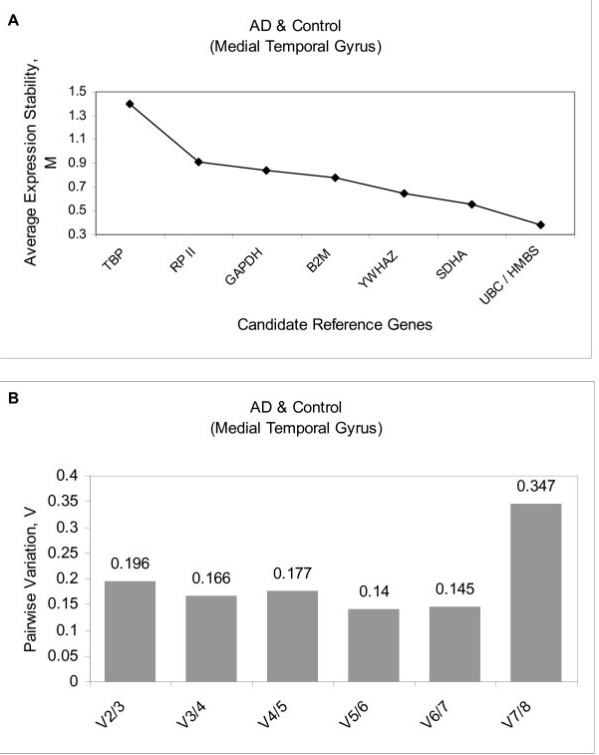
**Gene expression stability and pairwise variation of the candidate reference genes using geNorm analysis**. **A **– Expression stability plot showing the average expression stability (M) for the remaining genes following the sequential elimination of the least stable gene at each round. Least stable (left) and the two most stable (right). **B **– Pairwise variation analysis to determine the optimal number of reference genes for use in RT qPCR data normalization.

Analysis of data for the medial temporal gyrus of the control and AD subjects using geNorm produced a plot indicating the average expression stability of the remaining candidate reference genes in each round of the analysis (Figure 2A), ranking the candidate genes from least stable to the two most stable genes (Table 3A). Evaluation of the AD and control medial temporal gyrus group revealed a decrease in the pairwise variation with the inclusion of a fourth gene (compare V_2/3 _with V_3/4_). Although the pairwise variation at V_3/4 _(0.166) does not cross the threshold of 0.15, it can be seen from the increase in pairwise variation at V_4/5 _that the addition of a fifth gene was of no benefit (Figure 2B). Therefore, for data normalisation of this subject group, it would be appropriate to use UBC, HMBS, SDHA and YWHAZ as reference genes (denoted by an asterisk in Table 3A).

**Table 3:** Ranking of candidate reference genes in order of stability

**A**		
**MEDIAL TEMPORAL GYRUS**		
**AD + Controls**	**PD + Controls**	**DLB + Controls**

*UBC/*HMBS	*GAPDH/*SDHA	*B2M/*SDHA
*SDHA	*HMBS	*GAPDH
*YWHAZ	TBP	TBP
B2M	UBC	UBC
GAPDH	B2M	HMBS
RP II	RP II	RP II
TBP	YWHAZ	YWHAZ

**CEREBELLUM**		
**AD + Controls**	**PD + Controls**	**DLB + Controls**

*YWHAZ/*GAPDH	*HMBS/*GAPDH	*UBC/*GAPDH
*B2M	*SDHA	*B2M
SDHA	YWHAZ	HMBS
UBC	UBC	TBP
HMBS	TBP	RP II
TBP	RP II	SDHA
RP II	B2M	YWHAZ

**B**		

**GROUPED CEREBELLUM + MEDIAL TEMPORAL GYRUS**		
**AD + Controls**	**PD + Controls**	**DLB + Controls**

*B2M/*GAPDH	*RP II/*B2M	*UBC/*GAPDH
*SDHA	*UBC	*B2M
*UBC	*TBP	HMBS
*HMBS	SDHA	RP II
RP II	GAPDH	TBP
YWHAZ	HMBS	SDHA
TBP	YWHAZ	YWHAZ

Candidate references genes were ranked in order of stability for each group, with the two most stable genes at the top and the least stable at the bottom. An * indicates the set of reference genes which should be employed for the normalization of RT qPCR data for each group as determined from analysis of the pairwise variation.

Similar analysis of the medial temporal gyrus from controls and PD subjects ranked the candidate genes from the two most stable (GAPDH and SDHA) to the least stable (YWHAZ) (Additional File 2B). Use of the pairwise variation for the determination of the optimal number of control genes to be included indicated V_2/3 _= 0.138, below the threshold of 0.15. Therefore, although the inclusion of a fourth gene decreases the pairwise variation to 0.113, the use of three reference genes should be adequate for this group (Additional File 3B and Table 3A).

In the medial temporal gyrus of the DLB and control group, B2M and SDHA were found to be the most stable, with YWHAZ the least (Additional Figure 2C and Table 3A). As in the previous group, V_2/3 _falls below the threshold of 0.15, therefore the use of three reference genes (B2M, SDHA and GAPDH) should be sufficient (Additional File 3C and Table 3A).

The stability of each of the candidate reference genes was determined in cerebellum tissue of each subject grouping (as described above for the medial temporal gyrus), with a different rank order of the candidate reference genes being found for each group (Additional File 4 and Table 3B). Analysis of the pairwise variation with the inclusion of each additional gene was carried out to determine the optimal number of reference genes required for each group (Additional File 5and Table 3B).

A summary of the gene stability results for each subject grouping is presented in Table 3A &3B, with the optimum number of genes for each (as indicated by geNorm analysis) being indicated with asterisks. Some similarities in the ranking of candidate gene stability were observed between the medial temporal gyrus of each disease state with the cerebellum of the same group.

The stability of the candidate reference genes was also examined across a larger grouping that combined the cerebellum and medial temporal gyrus of each subject group. When considering genes for data normalization from cerebellum and medial temporal gyrus together, of both control and AD subjects, the candidate reference genes were ranked in order of stability from the most stable to the least stable (Additional File 6A). Calculation of the pairwise variation for the inclusion of each additional gene indicated five genes as the number of reference genes required to reach the threshold of 0.15 (Additional File 7A). Therefore, employing B2M, GAPDH, SDHA, UBC and HMBS as reference genes for data normalization would be necessary in the medial temporal gyrus and cerebellum of the control and AD subject group.

Similar analysis for the control and PD grouped subjects showed a gradual decrease in pairwise variation to V_6/7 _(Additional File 7B), although the inclusion of four genes produced a pairwise variation of 0.154, just over the threshold, indicating that four genes may be sufficient.

Analysis of the pairwise variation in DLB and control grouping demonstrated a V_2/3 _= 0.135; therefore although the inclusion of a fourth gene was found to improve the pairwise variation (Additional File 7C), the use of three reference genes (UBC, GAPDH, B2M) for normalisation of data in this group would be adequate.

As expected, due to the increased number of samples in these analyses, the level of variation increased, and hence additional reference genes would be required for accurate data normalization. Five, four and three reference genes were found to be the optimal number required for the cerebellum and medial temporal gyrus of the controls and AD subjects, controls and PD subjects, and controls and DLB subject groupings respectively (Table 3B).

In order to substantiate the findings from the geNorm analysis, our data was also analysed using an alternative application, NormFinder [15]. In addition to estimating the overall expression variation of the candidate reference genes, NormFinder also estimates the variation between sample subgroups. The reference gene stability results generated by NormFinder are presented in Additional File 8 for comparison.

## Discussion

As far as we are aware, this is the first study of the expression stability of candidate reference genes in post mortem brain tissue from individuals with AD, PD, DLB, and controls using geNorm analysis. This study identified groups of genes suitable for accurate normalization of RT qPCR data in the sample subsets. An overlap in the most suitable reference genes can be seen between disease states; for example, GAPDH was one of the most stable genes in the cerebellum of all disease states examined. This is in agreement with the work of Grunblatt *et al*. who found GAPDH to be suitable for RT qPCR data normalisation in the substantia nigra pars compacta of Parkinson's subjects [27]. B2M was found to be one of the most stable genes in the cerebellum for the AD, DLB (with control) groups. However, B2M was found to be the least stable in the PD and control cerebellum. This emphasises the fact that having a gene that is stable in one disease state does not mean the same gene will be stable in a different disease.

The top two most stable genes identified by Normfinder were also in the set recommended by geNorm, for AD, PD, and DLB temporal cortex (with controls), and PD, and DLB cerebellum. In the AD and control cerebellum, Normfinder identified UBC and SDHA as the top two stable genes, whereas geNorm identified YWAZ, GAPDH and B2M. This may indicate that expression of all these genes were stable enough to represent viable reference genes in the study.

There are a number of limitations to the present study that can be addressed in future work. One relates to the issue of RNA quality, as degradation of RNA can introduce error in this type of study, particularly if the extent of degradation varies between control and study groups [23]. This can be a particular problem in post mortem brain tissue obtained naturalistically. We attempted to maintain RNA integrity by using samples that were snap frozen following a short PMD, handling tissue on dry ice to avoid freeze-thawing, and including RNase inhibitors during extraction. Despite this, sample RINs were not ideal in this study, but importantly, they did not differ between control and disease groups. In addition, several practices were adopted to minimise the effect which any RNA degradation might have had on RT-qPCR assays. In the RT reactions, random nonomer primers were included in addition to the oligo dT primer, ensuring RT reaction priming at random sites along the RNA strand, in addition to being primed from the polyA tail, and minimising the possible influence of RNA degradation. A recent investigation of the effect of RNA integrity (using RIN) indicated that while a value of 5 or above was desirable for RT-qPCR, RINs (ranging from 1–10) did not affect PCR efficiency, and did not correlate with normalised data [26]. The chances of an amplicon spanning a break in the RNA increase dramatically with increasing size, and the importance of using short amplicons is highlighted in this study, which found that amplicons > 400 bp were strongly dependent on RNA integrity, while shorter products (70 – 250 bp) were 'independent' of RNA quality [26]. A separate study of post mortem brain RNA integrity and RT-qPCR assay data revealed an effect when data was normalised to β-actin (171 bp amplicon), and no effect when using another reference gene, β-glucuronidase with a shorter amplicon (81 bp) [23]. Our study used short amplicons throughout, of 127 bp or less. The lack of correlation observed between RIN and Q for any of the genes analysed in the present study, in either cerebellar or medial temporal gyrus tissue, indicates that these measures were successful.

We hypothesised that differential candidate reference gene expression may be observed in regions with different degrees of involvement in the neurodegenerative process. Since the temporal cortex is one of the earliest affected areas in AD, the increased variation of gene expression in the medial temporal gyrus of the AD subjects relative to the corresponding controls might have been expected (Fig 1E & F). Conversely, the cerebellum is thought to be one of the least affected areas of the AD brain and one would therefore expect it to display similar properties to that of the cerebellum from control subjects. However, our data shows a slight increase in the variation of gene expression between the cerebellum of AD subjects and that of the controls. This may be an indication that, although the cerebellum appears relatively unaffected by AD, some cerebellar pathology is present as previously described in the literature [28-30]. Interestingly, the observed range of expression of the genes in both the cerebellum and the medial temporal gyrus of the control subjects is generally much smaller than the range of expression of the same genes in the disease subjects. This observation again strengthens the argument for the need to validate reference genes for use in any given disease state.

## Conclusion

As proposed by Vandesompele *et al*. [17], we have identified several distinct sets of genes appropriate for RT qPCR data normalization in AD, PD and DLB post mortem brain tissue. Table 3A &3B indicate the appropriate reference genes from our panel for use in each disease state. This study has provided further evidence that candidate reference genes will not necessarily maintain their validity across differing brain regions or disease states, and that employing a single gene for data normalization would be inadequate.

## Methods

### Reference Genes

Potential candidate reference genes were identified from a search of the relevant literature, particularly relating to reference genes previously employed in neurodegenerative diseases. As can be seen in Table 1, the genes included in the study ranged from traditional, commonly used reference genes such as GAPDH to less well known genes such as HMBS, spanning a range of cellular functions. In some earlier studies of gene expression stability, ribosomal subunit RNAs were excluded from the gene panels since only oligo(dT) primers were employed in the RT [7]. Due to large differences in the expression levels between reference genes and possible target genes, we elected to exclude certain previously used genes, such as r18S, due their high levels of expression. Such high abundance relative to a target can make the subtraction of baseline values in the RT qPCR data analysis difficult [17]. However, since a modified RT protocol (described below) utilising both oligo(dT) and random monomer primers was employed, RP II was included.

β-actin has previously been employed as a reference gene in gene expression studies in substantia nigra of PD brain [27]. However, recent work by Barrachina *et al*. [23] indicated that β-actin was more susceptible to the effects of RNA degradation than other reference genes they examined, therefore we chose to exclude it.

### Tissue Samples

Human post-mortem brain tissue was obtained via the rapid autopsy program of The Netherlands Brain Bank (NBB), (Netherlands Institute for Neuroscience, Amsterdam, The Netherlands) which supplies post-mortem specimens from clinically well documented and neuropathologically confirmed cases. Autopsies are performed on donors from whom written informed consent has been obtained from either the donor or direct next of kin. The work of the NBB abides by the Ethical code of conduct approved by the ethics committee. Information is available for all donors to allow matching for various ante- and post-mortem factors. Ante-mortem information includes age, sex, agonal state, date and time of death and medication [31,32]. Post-mortem information includes post-mortem delay (PMD), pH of cerebrospinal fluid (CSF), and tissue storage time.

Tissue from the medial temporal gyrus and cerebellum were obtained from 5 control subjects with no history of neurological or psychiatric disorders and from 6 AD, 6 PD, and 6 DLB subjects. Tissue was dissected, snap frozen under liquid nitrogen, and stored at -70°C. Details of diagnosis, sex, age, Braak staging, PMD, and CSF pH for the subjects are presented in Table 4.

**Table 4:** Main clinical and neuropathological data for subjects

**Diagnosis**	**Sex**	**Age**	**Braak Stage**	**PM Delay (min)**	**CSF pH**
**AD**	f	81	4	193	6.54
	f	85	4	220	6.59
	f	77	5	215	6.67
	m	95	5	185	6.4
	m	58	6	385	6.42
	f	87	6	300	6.66

Mean ± S.D.		80.50 ± 11.49		249.67 ± 71.09	6.55 ± 0.11

**PD**	m	75	n/a	255	6.42
	f	83	3	290	6.76
	f	80	3	480	6.85
	m	82	3	425	7.64
	f	80	3	255	6.50
	f	84	1	370	6.50

Mean ± S.D.		80.67 ± 2.92		345.83 ± 86.09	6.78 ± 0.41

**DLB**	f	79	1	200	6.4
	m	74	1	255	6.12
	m	81	2	345	6.63
	m	86	2	472	n/a
	f	79	3	235	6.72
	m	95	2	270	6.6

Mean ± S.D.		82.33 ± 6.67		296.17 ± 90.07	6.49 ± 0.21

**Controls**	f	69	1	375	6.59
	f	72	1	405	6.52
	m	79	1	320	6.72
	m	83	1	275	6.49
	f	88	2	340	n/a

Mean ± S.D.		78.20 ± 6.97		343.00 ± 44.79	6.58 ± 0.09

Unpaired t-tests were used to verify that the AD, PD, DLB and control groups were matched for age, post-mortem (PM) delay and pH of CSF.

### Extraction of Total RNA and cDNA Synthesis

Care was taken to prevent RNA degradation by employing good molecular biology practices. These included the use of gloves at all times, the use of nuclease free molecular biology grade water (Eppendorf) for all buffers, and the cleaning of all working surfaces with RNase Away (Molecular BioProducts) prior to working.

Tissue samples (~100 mg) were cut from the snap frozen post-mortem samples on dry ice, transferred immediately to QIAzol lysis reagent (Qiagen), which facilitates lysis of fatty tissues and inhibit RNases, and then homogenised using an Ultra-Turrax T25 homogeniser. Total RNA was extracted using an RNeasy^® ^Lipid Tissue Mini Kit (Qiagen), which includes DNase treatment to degrade genomic DNA. Total RNA in each sample was quantified using the (fluorescent) RNA specific Ribogreen^® ^assay, which employs an RNA standard curve (Molecular Probes, Invitrogen). Purified total RNA was heat denatured (2 min @ 70°C). RNA integrity was determined using RNA 6000 Nano Labchips^® ^in an Agilent 2100 Bioanalyzer following the manufactures protocol. The concentration of RNA obtained from the tissue samples varied from 0.19 – 1.16 μg/μl and was adjusted to 0.1 μg/μl using nuclease-free water. 2 μg RNA was used in each 20 μl reverse transcription (RT) reaction. The RT reactions were performed with an Omniscript^® ^Reverse Transcription Kit (Qiagen) according to the manufacturers protocol in which random nonomer primers at a final concentration of 10 μM were added to the master mix, in addition to the oligo dT primers.

### Real-Time PCR

RT qPCR reactions were carried out for all genes of interest in each sample using cDNA specific TaqMan^® ^Gene Expression Assays on an ABI 7500 Real-Time PCR System (Applied Biosystems). In each 25 μl TaqMan^® ^reaction, 10 μl cDNA (corresponding to the cDNA reverse transcribed from approximately 10 ng RNA) was mixed with 1.25 μl TaqMan^® ^Gene Expression Assay and 12.5 μl TaqMan^® ^Universal PCR Master Mix (Applied Biosystems) and 1.25 μl H_2_0. This allowed for the consistent use of standardised thermal cycling conditions: 95°C for 10 min, followed by 40 cycles of 95°C for 15 sec and 60°C for 1 min (note that the 50°C for 2 min step was omitted since AmpErase UNG was not included in the reaction mixture) which were found to give efficiencies > 92% (see below). Unless otherwise stated, all RT qPCR reactions were run in duplicate.

No template control reactions were included in each assay run. The Taqman^® ^Gene Expression assays employed are cDNA specific assays, and are therefore unable to detect genomic DNA if present.

### PCR Efficiency

A 10-fold dilution series was created from a random pool of cDNA from our sample group (including age matched controls, AD, PD and DLB patient samples) ranging from ×10 dilution to ×100000 dilution. Triplicate RT qPCR reactions were carried out as described above for each gene at each dilution. Mean cycle threshold (C_t_) values for each dilution were plotted against the log_10 _of the cDNA input for each gene to generate efficiency plots. The reaction efficiency for each gene assay was calculated using the following equation: E = 10^(-1/slope) ^where E is the reaction efficiency and 'slope' is the slope of the line generated in the efficiency plots.

### Data Analysis

The mean C_t _values of the replicates for each sample were transformed into raw, non-normalised quantities (Q) using the comparative ΔC_t _method by the equation Q = E^ΔCt ^where E is the reaction efficiency for each gene assay in question and ΔC_t _= min C_t _– sample C_t_, where min C_t _is the lowest C_t _value over a range of samples for a given assay, and sample C_t _is the C_t _value of the sample being transformed. The expression data was analysed using the geNorm algorithm [33] which determines a reference gene stability factor (M), defined as the average pairwise variation of a particular gene compared with all of the other candidate reference genes [17]. Hence, a lower value of M indicates higher stability of the reference gene.

## Authors' contributions

DC conceived the study design, performed the experimental procedures, carried out the analysis and interpretation of data, and drafted the manuscript. SB and JQ assisted in the preparation of RNA and cDNA from the post-mortem samples and participated in the RT qPCR. SM assisted in the preparation of RNA from the post-mortem samples. RR provided the post-mortem tissue collected from Brain Bank donors and supplied all autopsy data used in this manuscript as well as the clinical and neuropathological documentation of the samples. GBI participated in the study design, the analysis and interpretation of data and carried out critical revision of the manuscript. JJ participated in the conception and design of the study, helped to draft the manuscript and supervised the study.

## Supplementary Material

Additional file 1 **RT qPCR Efficiency Plots **Mean cycle threshold (C_t_) values for each dilution were plotted against the log_10 _of the cDNA input for each gene to generate efficiency plots. The reaction efficiency for each gene assay was calculated using the following equation: E = 10^(-1/slope) ^where E is the reaction efficiency and 'slope' is the slope of the line generated in the efficiency plots. Click here for file

Additional file 2 **Stability of candidate reference genes in medial temporal gyrus**. Expression stability plot showing the average expression stability (M) for the remaining genes following the sequential elimination of the least stable gene at each round in the medial temporal gyrus of control and PD subjects (A), and control and DLB subjects (B). Least stable (left) and the two most stable (right). Click here for file

Additional file 3 **Pairwise variation of the candidate reference genes in medial temporal gyrus**. Pairwise variation analysis to determine the optimal number of reference genes for use in RT qPCR data normalization from cerebellum of control and PD subjects (A), and control and DLB subjects (B). To determine the optimal number of genes required for geometric mean normalization, geNorm calculates the pairwise variation (V_n_/V_n+1_) between sequential normalization factors (NF) (NF_n _and NF_n+1_). A large variation indicates that the gene included at that stage has a significant effect and should therefore be included for normalization. Click here for file

Additional file 4 **Stability of candidate reference genes in cerebellum**. Expression stability plot showing the average expression stability (M) for the remaining genes following the sequential elimination of the least stable gene at each round in cerebellum of control and AD subjects (A), control and PD subjects (B), and control and DLB subjects (C). Least stable (left) and the two most stable (right). Click here for file

Additional file 5 **Pairwise variation of the candidate reference genes in cerebellum**. Pairwise variation analysis to determine the optimal number of reference genes for use in RT qPCR data normalization from cerebellum of control and AD subjects (A), control and PD subjects (B), and control and DLB subjects (C). To determine the optimal number of genes required for geometric mean normalization, geNorm calculates the pairwise variation (V_n_/V_n+1_) between sequential normalization factors (NF) (NF_n _and NF_n+1_). A large variation indicates that the gene included at that stage has a significant effect and should therefore be included for normalization. Click here for file

Additional file 6 **Stability of candidate reference genes in cerebellum and medial temporal gyrus**. Expression stability plot showing the average expression stability (M) for the remaining genes following the sequential elimination of the least stable gene at each round in cerebellum and medial temporal gyrus of control and AD subjects (A), control and PD subjects (B), and control and DLB subjects (C). Least stable (left) and the two most stable (right). Click here for file

Additional file 7 **Pairwise variation of the candidate reference genes in cerebellum and medial temporal gyrus**. Pairwise variation analysis to determine the optimal number of reference genes for use in RT qPCR data normalization from cerebellum of control and AD subjects (A), control and PD subjects (B), and control and DLB subjects (C). To determine the optimal number of genes required for geometric mean normalization, geNorm calculates the pairwise variation (V_n_/V_n+1_) between sequential normalization factors (NF) (NF_n _and NF_n+1_). A large variation indicates that the gene included at that stage has a significant effect and should therefore be included for normalization. Click here for file

Additional file 8 **Stable Reference Genes as Determined by NormFinder**. The expression data for each of the candidate reference genes was analysed using the Excel based application 'NormFinder'. Click here for file
