# Supplementary material for: Identification of valid reference genes for the normalization of RT qPCR gene expression data in human brain tissue
Source: BMC Mol Biol. 2008 May 6;9:46. doi: 10.1186/1471-2199-9-46 (PMC2396658; doi:10.1186/1471-2199-9-46)
Supplement: Additional file 8 — Stable Reference Genes as Determined by NormFinder. The expression data for each of the candidate reference genes was analysed using the Excel based application 'NormFinder'. [file 1471-2199-9-46-S8.doc]

## Additional Table 1 – NormFinder Analysis

| **Brain Region** | **Sample Groups** | **Best Gene** | **Stability Value for Best Gene** | **Best comb of 2 Genes** | **Stability Value for best comb of 2 genes** |
| --- | --- | --- | --- | --- | --- |
| **Medial Temporal Gyrus** | AD + Ctrl | SDHA | 0.015 | HMBS / SDHA | 0.015 |
|  | PD + Ctrl | GAPDH | 0.028 | GAPDH / SDHA | 0.023 |
|  | DLB + Ctrl | SDHA | 0.033 | B2M / SDHA | 0.035 |
| **Cerebellum** | AD + Ctrl | SDHA | 0.015 | UBC / SDHA | 0.012 |
|  | PD + Ctrl | SDHA | 0.046 | GAPDH / SDHA | 0.043 |
|  | DLB + Ctrl | UBC | 0.027 | UBC / B2M | 0.026 |
| **Cerebellum & Medial Temporal Gyrus** | AD + Ctrl | SDHA | 0.012 | UBC / SDHA | 0.020 |
|  | PD + Ctrl | SDHA | 0.021 | UBC / SDHA | 0.020 |
|  | DLB + Ctrl | UBC | 0.016 | UBC / SDHA | 0.013 |
