# Supplementary material for: Identification of valid reference genes for the normalization of RT qPCR gene expression data in human brain tissue
Source: BMC Mol Biol. 2008 May 6;9:46. doi: 10.1186/1471-2199-9-46 (PMC2396658; doi:10.1186/1471-2199-9-46)
Supplement: Supplementary file 2 [file 1471-2199-9-46-S1.doc]

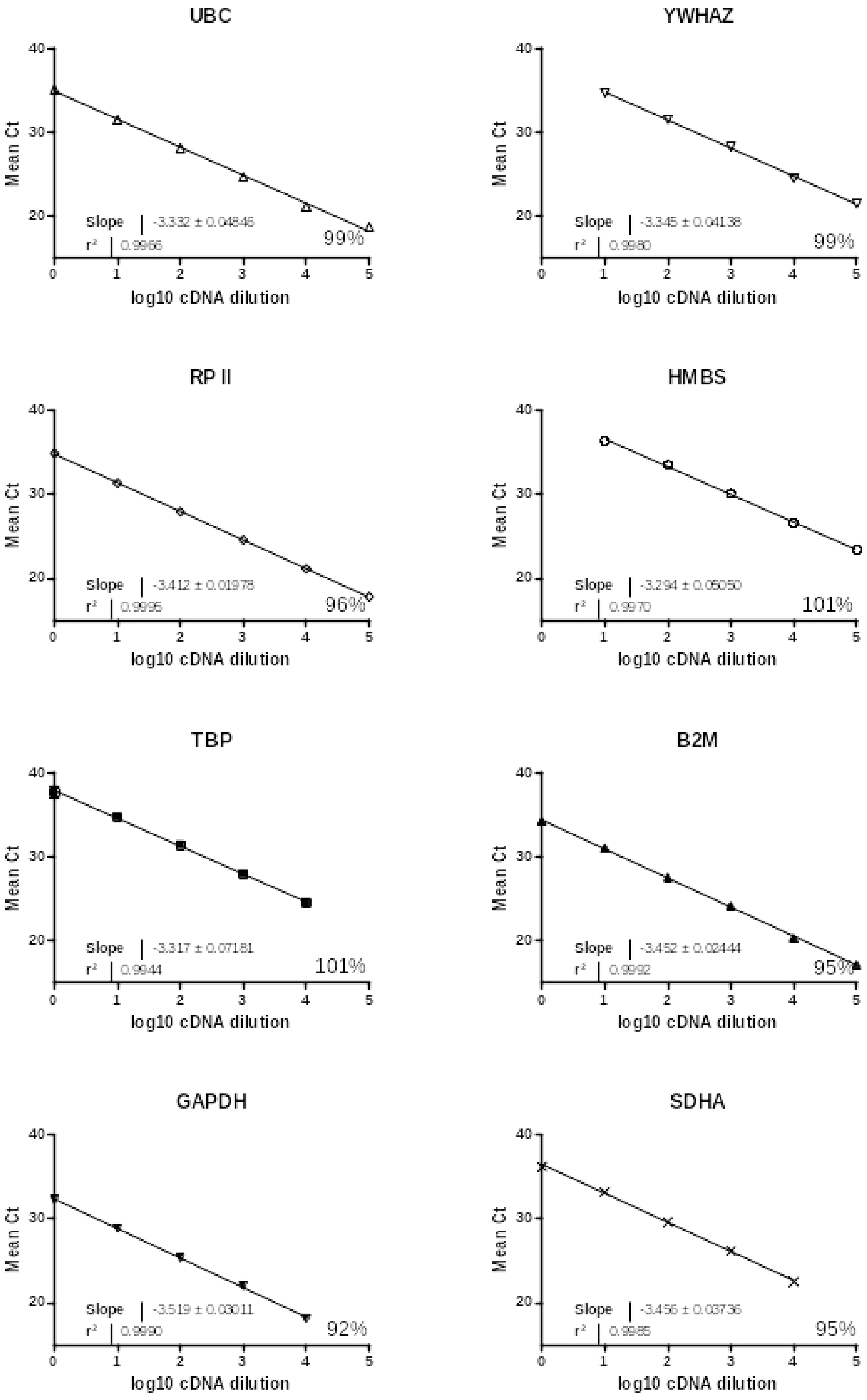
UBC
Slope -3.332 ± 0.04846
r² 0.9966
99%
YWHAZ
Slope -3.345 ± 0.04136
r² 0.9980
99%
RP II
Slope -3.412 ± 0.01978
r² 0.9995
96%
HMBS
Slope -3.294 ± 0.05050
r² 0.9970
101%
TBP
Slope -3.317 ± 0.07181
r² 0.9944
101%
B2M
Slope -3.452 ± 0.02444
r² 0.9992
95%
GAPDH
Slope -3.519 ± 0.03011
r² 0.9990
92%
SDHA
Slope -3.456 ± 0.03736
r² 0.9985
95%
Mean Ct
log10 cDNA dilution
