# Supplementary material for: Identification of valid reference genes for the normalization of RT qPCR gene expression data in human brain tissue
Source: BMC Mol Biol. 2008 May 6;9:46. doi: 10.1186/1471-2199-9-46 (PMC2396658; doi:10.1186/1471-2199-9-46)
Supplement: Supplementary file 3 [file 1471-2199-9-46-S2.doc]

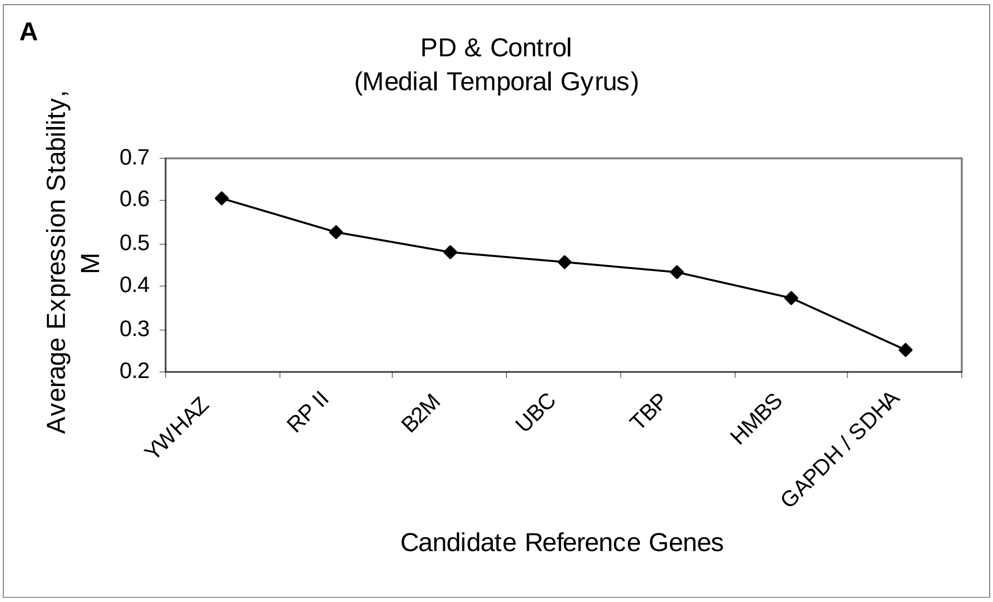
A
PD & Control
(Medial Temporal Gyrus)
Average Expression Stability, M
0.7
0.6
0.5
0.4
0.3
0.2
YWHAZ
RP II
B2M
UBC
TBP
HMBS
GAPDH / SDHA
Candidate Reference Genes

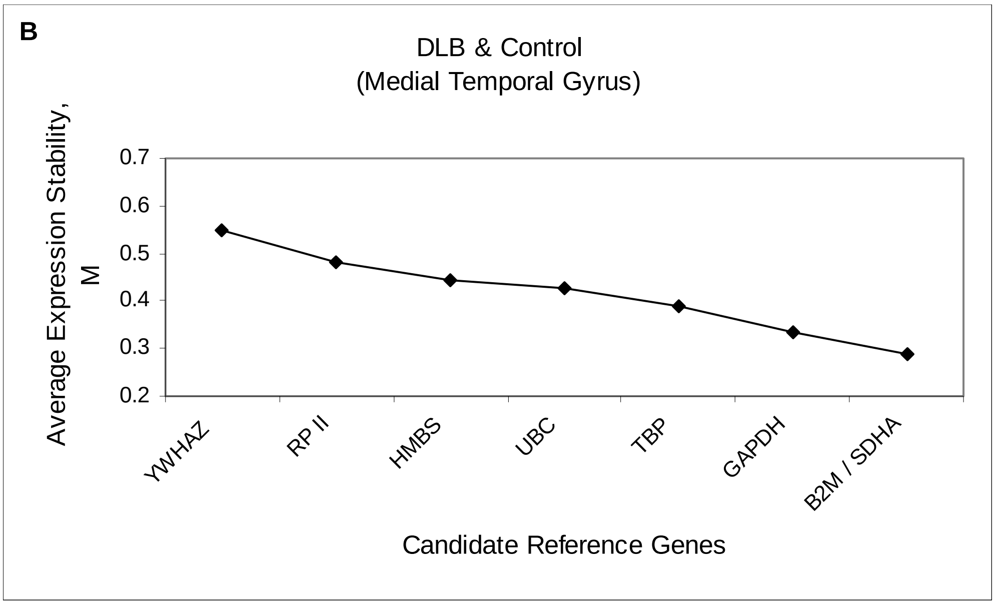
B
DLB & Control
(Medial Temporal Gyrus)
Average Expression Stability, M
0.7
0.6
0.5
0.4
0.3
0.2
YWHAZ
RP II
HMBS
UBC
TBP
GAPDH
B2M / SDHA
Candidate Reference Genes
