# Supplementary material for: Identification of valid reference genes for the normalization of RT qPCR gene expression data in human brain tissue
Source: BMC Mol Biol. 2008 May 6;9:46. doi: 10.1186/1471-2199-9-46 (PMC2396658; doi:10.1186/1471-2199-9-46)
Supplement: Supplementary file 4 [file 1471-2199-9-46-S3.doc]

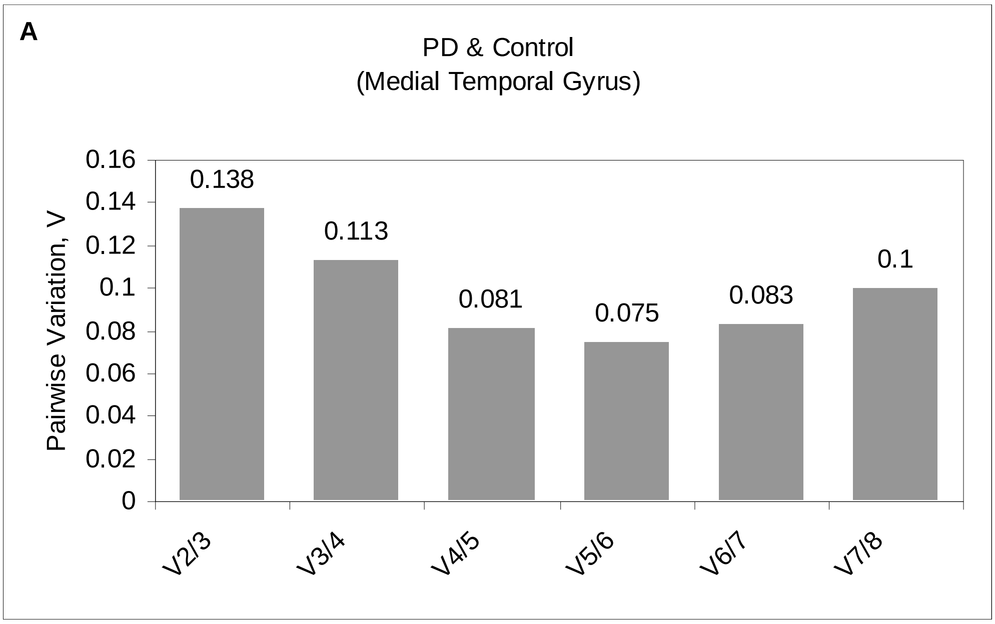
A
PD & Control
(Medial Temporal Gyrus)
Pairwise Variation, V
0.16
0.14
0.12
0.1
0.08
0.06
0.04
0.02
0
0.138
0.113
0.081
0.075
0.083
0.1
V2/3
V3/4
V4/5
V5/6
V6/7
V7/8

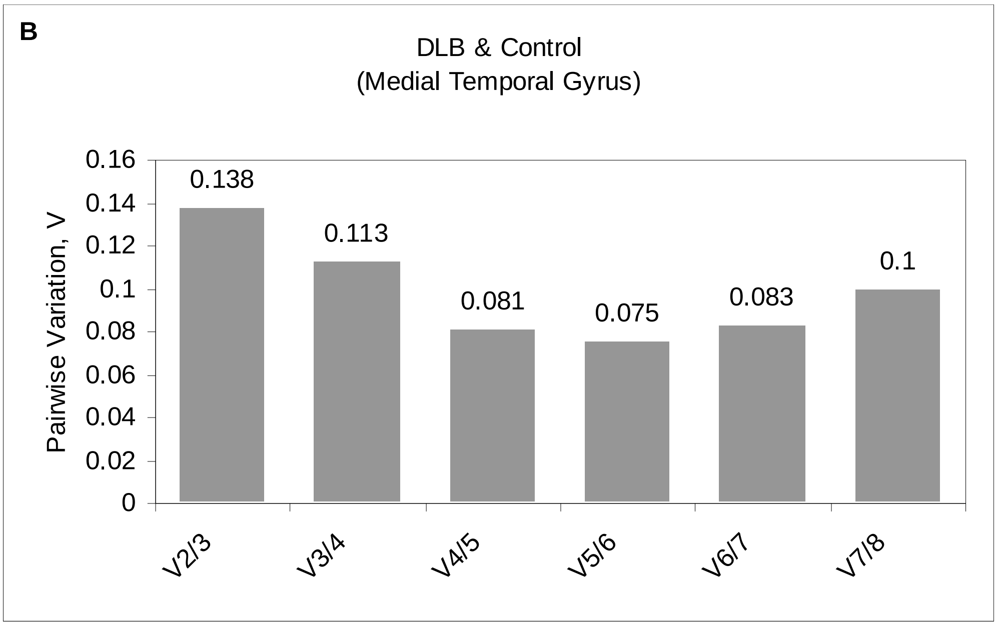
B
DLB & Control
(Medial Temporal Gyrus)
Pairwise Variation, V
0.16
0.14
0.12
0.1
0.08
0.06
0.04
0.02
0
0.138
0.113
0.081
0.075
0.083
0.1
V2/3
V3/4
V4/5
V5/6
V6/7
V7/8
