# Supplementary material for: Identification of valid reference genes for the normalization of RT qPCR gene expression data in human brain tissue
Source: BMC Mol Biol. 2008 May 6;9:46. doi: 10.1186/1471-2199-9-46 (PMC2396658; doi:10.1186/1471-2199-9-46)
Supplement: Supplementary file 5 [file 1471-2199-9-46-S4.doc]

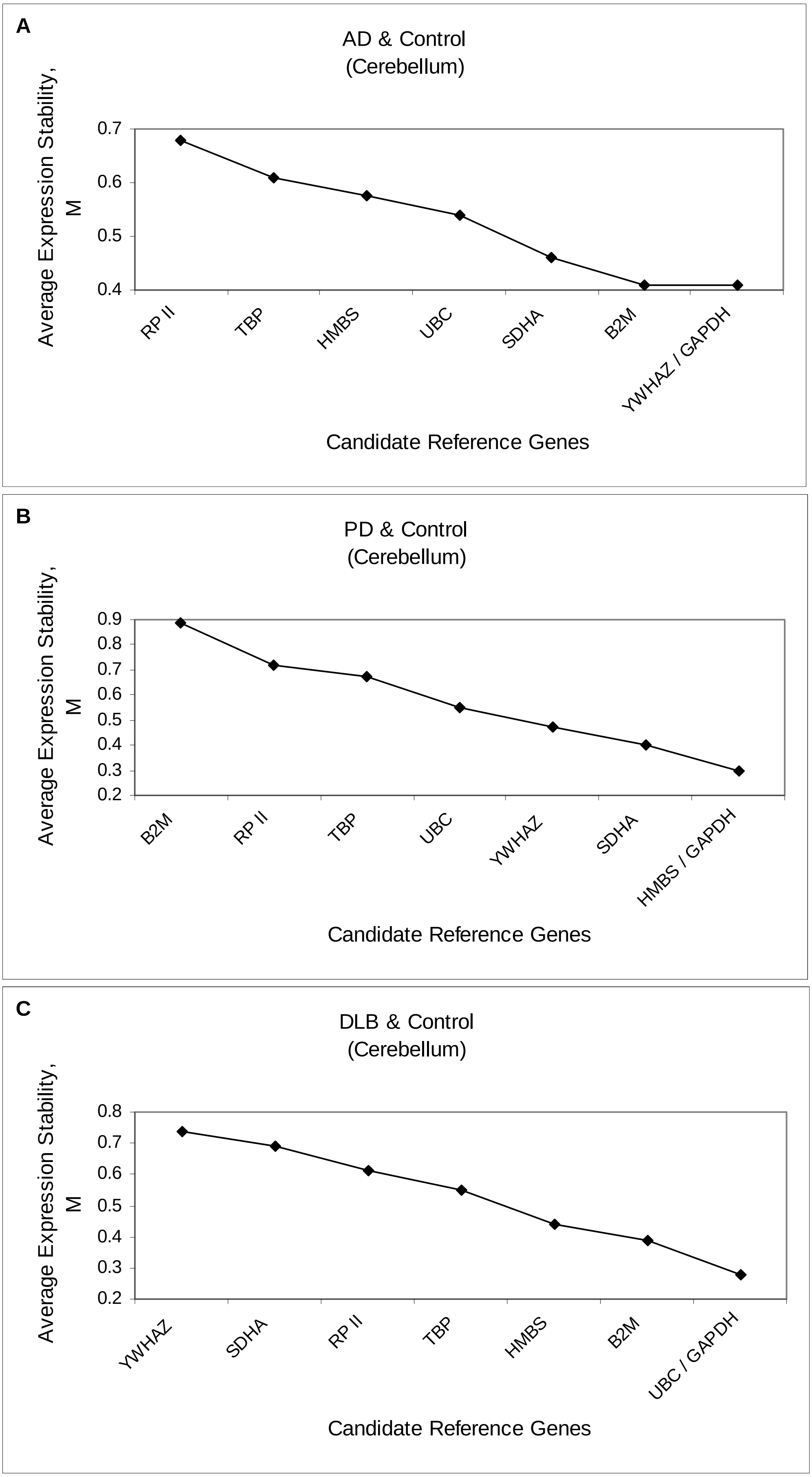
A
AD & Control
(Cerebellum)
Average Expression Stability, M
0.7
0.6
0.5
0.4
RP II
TBP
HMBS
UBC
SDHA
B2M
YWHAZ / GAPDH
Candidate Reference Genes
B
PD & Control
(Cerebellum)
Average Expression Stability, M
0.9
0.8
0.7
0.6
0.5
0.4
0.3
0.2
B2M
RP II
TBP
UBC
YWHAZ
SDHA
HMBS / GAPDH
Candidate Reference Genes
C
DLB & Control
(Cerebellum)
Average Expression Stability, M
0.8
0.7
0.6
0.5
0.4
0.3
0.2
YWHAZ
SDHA
RP II
TBP
HMBS
B2M
UBC / GAPDH
Candidate Reference Genes
