# Supplementary material for: Identification of valid reference genes for the normalization of RT qPCR gene expression data in human brain tissue
Source: BMC Mol Biol. 2008 May 6;9:46. doi: 10.1186/1471-2199-9-46 (PMC2396658; doi:10.1186/1471-2199-9-46)
Supplement: Supplementary file 6 [file 1471-2199-9-46-S5.doc]

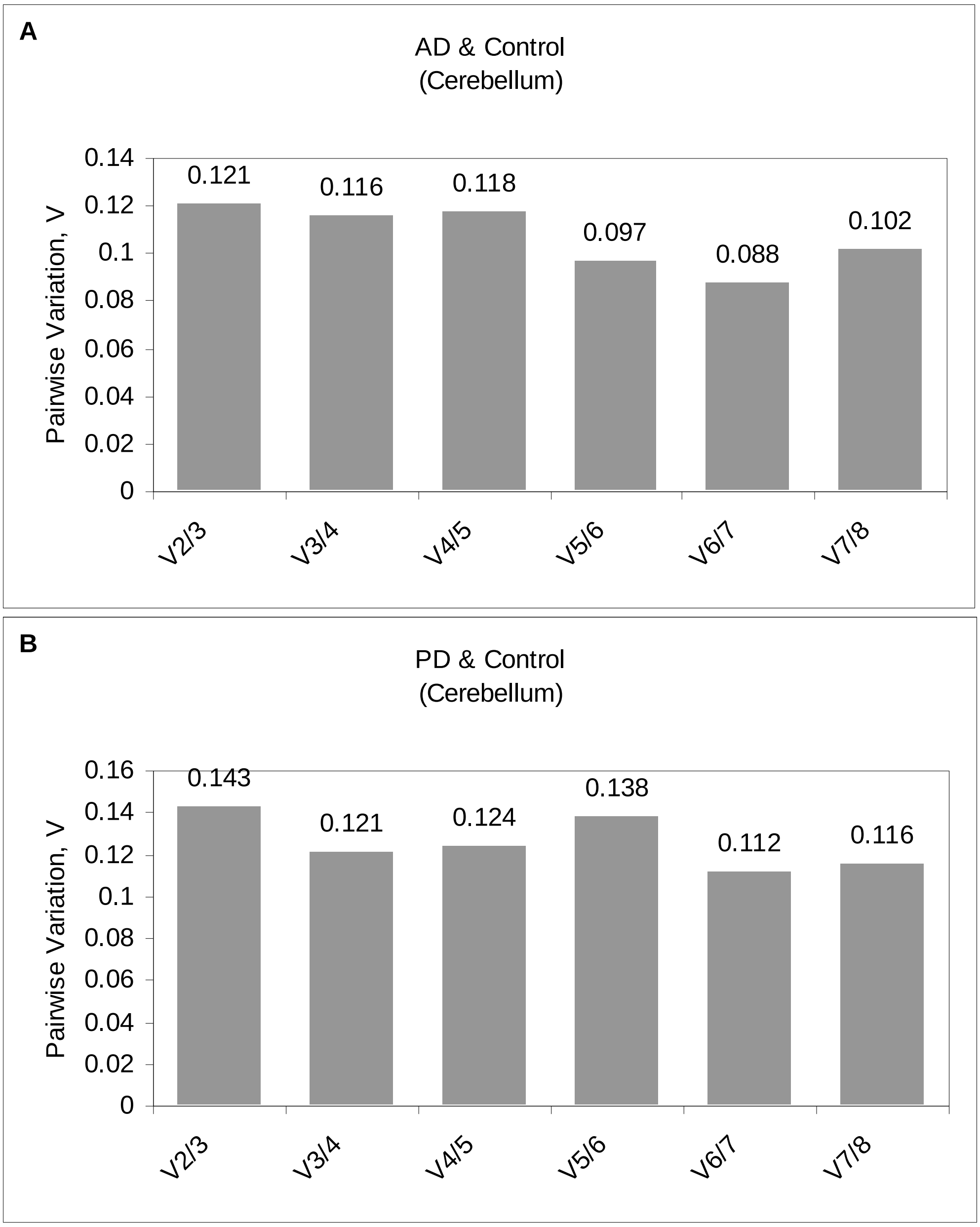
A
AD & Control
(Cerebellum)
Pairwise Variation, V
0.14
0.12
0.1
0.08
0.06
0.04
0.02
0
0.121
0.116
0.118
0.097
0.088
0.102
V2/3
V3/4
V4/5
V5/6
V6/7
V7/8
B
PD & Control
(Cerebellum)
Pairwise Variation, V
0.16
0.14
0.12
0.1
0.08
0.06
0.04
0.02
0
0.143
0.121
0.124
0.138
0.112
0.116
V2/3
V3/4
V4/5
V5/6
V6/7
V7/8

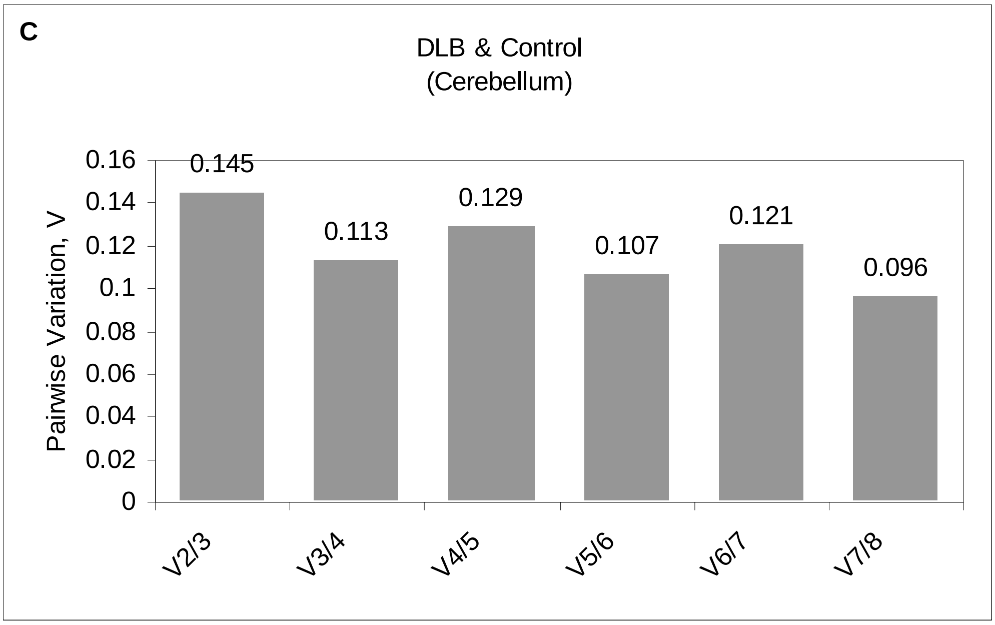
C
DLB & Control
(Cerebellum)
Pairwise Variation, V
0.16
0.14
0.12
0.1
0.08
0.06
0.04
0.02
0
0.145
0.113
0.129
0.107
0.121
0.096
V2/3
V3/4
V4/5
V5/6
V6/7
V7/8
