# Supplementary material for: Identification of valid reference genes for the normalization of RT qPCR gene expression data in human brain tissue
Source: BMC Mol Biol. 2008 May 6;9:46. doi: 10.1186/1471-2199-9-46 (PMC2396658; doi:10.1186/1471-2199-9-46)
Supplement: Supplementary file 7 [file 1471-2199-9-46-S6.doc]

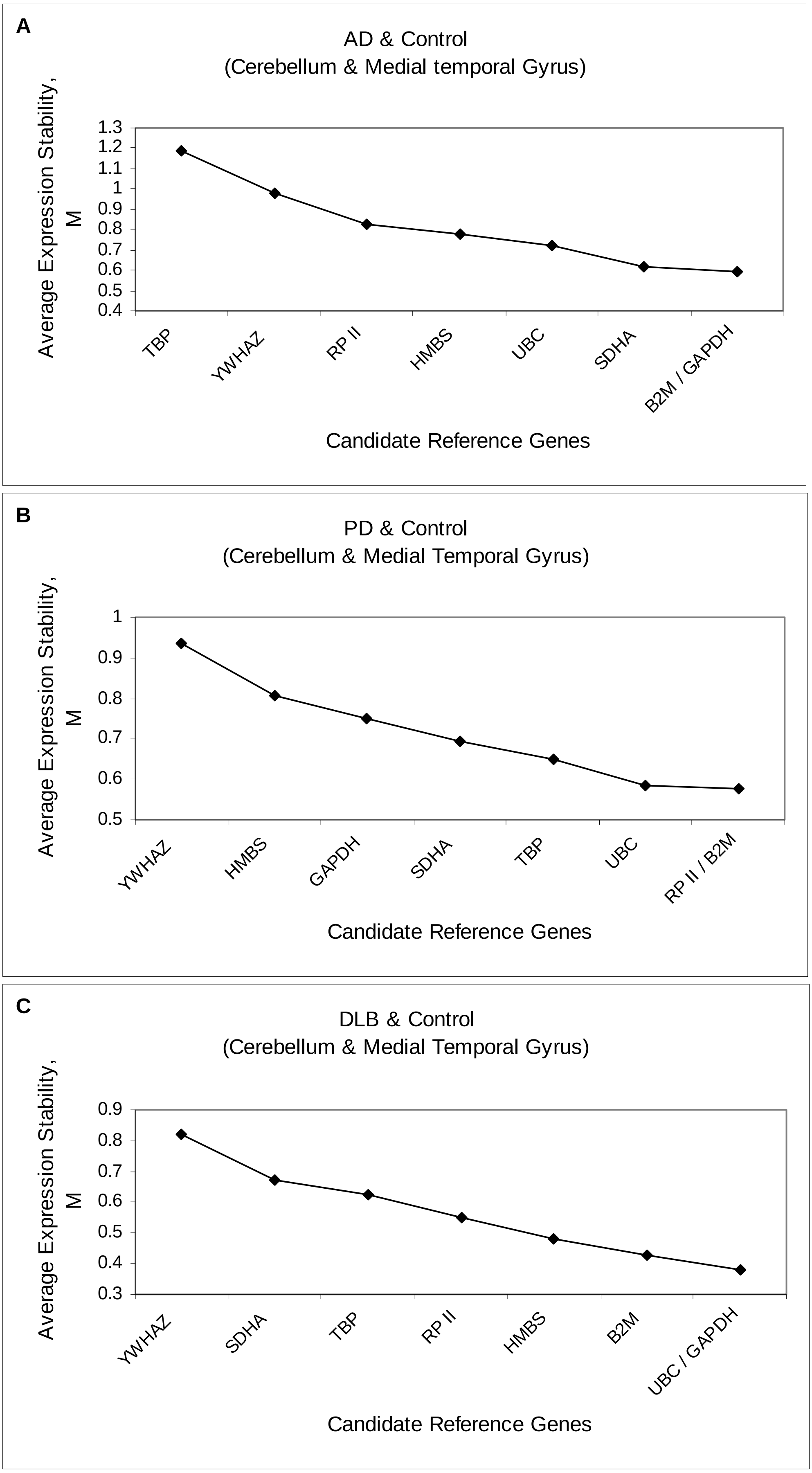
A
AD & Control
(Cerebellum & Medial temporal Gyrus)
Average Expression Stability, M
1.3
1.2
1.1
1
0.9
0.8
0.7
0.6
0.5
0.4
TBP
YWHAZ
RP II
HMBS
UBC
SDHA
B2M / GAPDH
Candidate Reference Genes
B
PD & Control
(Cerebellum & Medial Temporal Gyrus)
Average Expression Stability, M
1
0.9
0.8
0.7
0.6
0.5
YWHAZ
HMBS
GAPDH
SDHA
TBP
UBC
RP II / B2M
Candidate Reference Genes
C
DLB & Control
(Cerebellum & Medial Temporal Gyrus)
Average Expression Stability, M
0.9
0.8
0.7
0.6
0.5
0.4
0.3
YWHAZ
SDHA
TBP
RP II
HMBS
B2M
UBC / GAPDH
Candidate Reference Genes
