# Supplementary material for: Identification of valid reference genes for the normalization of RT qPCR gene expression data in human brain tissue
Source: BMC Mol Biol. 2008 May 6;9:46. doi: 10.1186/1471-2199-9-46 (PMC2396658; doi:10.1186/1471-2199-9-46)
Supplement: Supplementary file 8 [file 1471-2199-9-46-S7.doc]

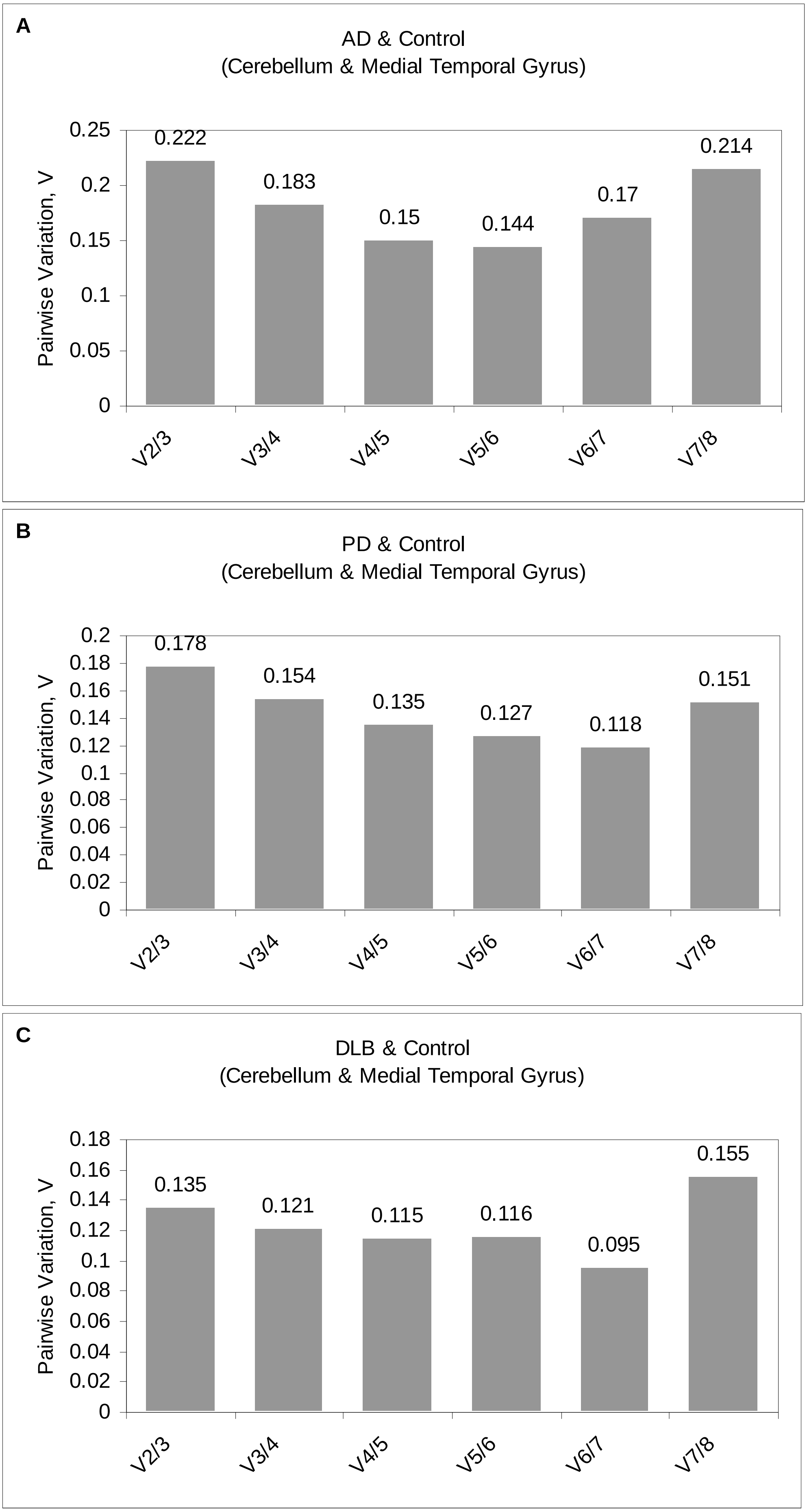
A
AD & Control
(Cerebellum & Medial Temporal Gyrus)
Pairwise Variation, V
0.25
0.2
0.15
0.1
0.05
0
0.222
0.183
0.15
0.144
0.17
0.214
V2/3
V3/4
V4/5
V5/6
V6/7
V7/8
B
PD & Control
(Cerebellum & Medial Temporal Gyrus)
Pairwise Variation, V
0.2
0.18
0.16
0.14
0.12
0.1
0.08
0.06
0.04
0.02
0
0.178
0.154
0.135
0.127
0.118
0.151
V2/3
V3/4
V4/5
V5/6
V6/7
V7/8
C
DLB & Control
(Cerebellum & Medial Temporal Gyrus)
Pairwise Variation, V
0.18
0.16
0.14
0.12
0.1
0.08
0.06
0.04
0.02
0
0.135
0.121
0.115
0.116
0.095
0.155
V2/3
V3/4
V4/5
V5/6
V6/7
V7/8
